# Estimation of glomerular filtration rate by standard renal CT imaging

**DOI:** 10.1186/s13244-026-02431-0

**Published:** 2026-10-14

**Authors:** Kristoffer Huitfeldt Sola, Helena Genberg, Joshua Webster-Ford, Anders Ullén, Torkel B. Brismar

**Affiliations:** 1 Division of Radiology, CLINTEC, Karolinska Institutet, Stockholm, Sweden; 2 Department of Transplantation Surgery, Karolinska University Hospital, Stockholm, Sweden; 3 Division of Transplantation Surgery, CLINTEC, Karolinska Institutet, Stockholm, Sweden; 4 Department of Pelvic Cancer, Genitourinary Oncology Division, Karolinska University Hospital, Stockholm, Sweden; 5 Department of Oncology-Pathology, Karolinska Institute, Stockholm, Sweden; 6 Department of Radiology, Karolinska University Hospital, Stockholm, Sweden

**Keywords:** Tomography, X-ray computed, Glomerular filtration rate, Kidney, Image processing, computer-assisted, Artificial intelligence

## Abstract

**Objectives:**

To develop and evaluate a method to predict glomerular filtration rate (GFR) from clinical renal CT.

**Materials and methods:**

Medical records of adult living kidney donors operated between 2013 and 2022 and patients considered for neoadjuvant cisplatin prior to cystectomy between 2019 and 2024 were retrospectively reviewed. Inclusion criteria were (1) renal CT obtained at 100 or 120 kVp before and 5–15 min after contrast administration, and (2) iohexol clearance test within 2 months of imaging. The iohexol clearance test was performed on the same day as the cystatin C and creatinine measurements. Estimated GFR values included cystatin C–based eGFR (eGFR_cystatin C_), creatinine-based eGFR using the Cockcroft–Gault formula (eGFR_Cockcroft-Gault_), and eGFR using the Revised Lund–Malmö formula (eGFR_LMR18_), which is based on both creatinine and cystatin C. Manual and automatic segmentations of the renal pelvis and bladder were performed in both the excretory and unenhanced phases.

**Results:**

In total, 139 patients were included in the analysis (mean age 52 ± 13 years; 60% women). Multivariate linear regression analysis combining the segmentation-derived variable with body surface area and age gave an adjusted R-square of 0.77 to mGFR_iohexol_ (*p* < 0.001). GFR_CT_ had the strongest relationship to mGFR_iohexol_ with a mean absolute error of 12.8 mL/min, compared with 15.6 mL/min for eGFR_Cockcroft-Gault_, 14.2 mL/min for eGFR_cystatin C_, and 13.0 mL/min for eGFR_LMR18_. Manual and automatic segmentations had similar performance. Patients evaluated for cisplatin treatment had greater variation in CT-estimated split renal function than donors.

**Conclusion:**

Renal function can be estimated with high precision from clinical renal CT.

**Key Points:**

***Question*** Accurate GFR assessment is critical, but current methods are either labor-intensive or imprecise. Can renal CT imaging provide a reliable alternative?

***Findings*** CT-derived eGFR showed the strongest correlation with iohexol clearance (R² = 0.77), with lower error compared to creatinine- and cystatin C–based estimates.

***Critical relevance statement*** Routine CT imaging can provide precise estimates of kidney function, supporting donor evaluations, chemotherapy dosing, and critical care decisions when accurate renal function is essential.

**Graphical Abstract:**

**Figure:**
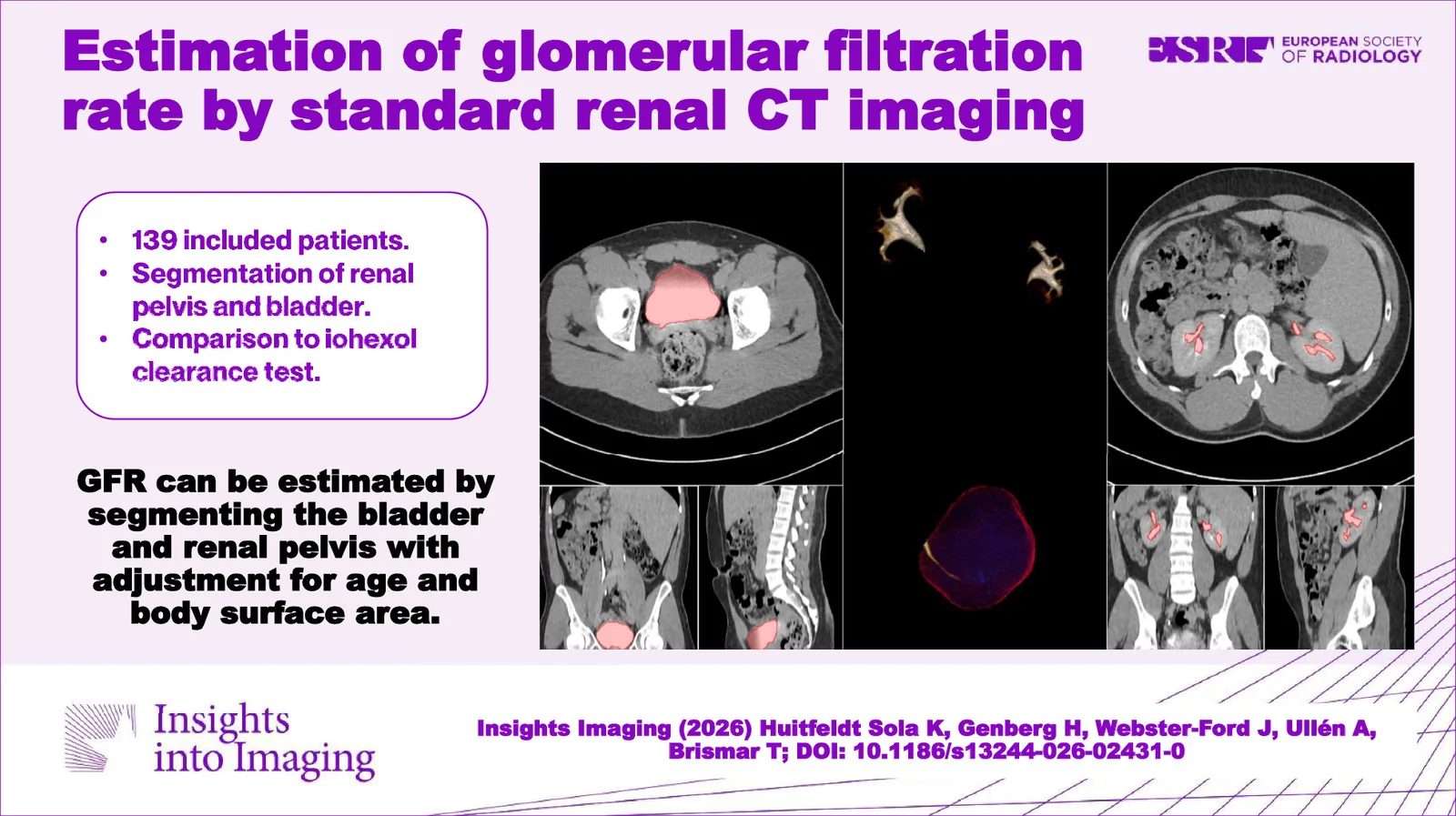

## Introduction

Glomerular filtration rate (GFR) describes the volume of fluid filtered through the kidneys per unit of time [1]. Accurate assessment of GFR is critical in a wide range of clinical settings, including the staging of chronic kidney disease, selection for nephrotoxic treatments, and organ transplantation planning.

Absolute GFR can be directly measured by evaluating the clearance of exogenous filtration markers. Although inulin clearance has historically been regarded as the reference standard for measured GFR (mGFR), its complexity has limited routine clinical use [2, 3]. Iohexol plasma clearance has therefore emerged as a practical and well-validated alternative. This test is regarded as the gold standard due to its independence from confounding factors such as muscle mass, inflammation, and protein intake [1]. However, it is labor-intensive, time-consuming, and costly, limiting its widespread use, particularly in time-sensitive settings [3].

Most clinical assessments rely on estimated GFR (eGFR) derived from endogenous markers, mainly serum creatinine or cystatin C [1]. These estimations are practical and highly accessible but suffer from considerable interindividual variability. Creatinine-based equations, such as Cockcroft–Gault and the Revised Lund–Malmö formula, are prone to inaccuracies from factors like muscle mass, protein intake, and physical activity [4]. Cystatin C–based estimates may improve precision but are influenced by systemic inflammation, thyroid function, adiposity, and steroid use [4]. Across commonly used methods, an error margin of up to ± 30% has been documented, with GFR and eGFR differing by more than 30% in approximately 20% of individuals, particularly at the extremes of renal function [5, 6].

Iohexol is also employed as a contrast medium (CM) during routine computed tomography (CT) scans [3]. This presents an opportunity to measure GFR non-invasively from CT imaging. An image-based method to extract GFR data from contrast-enhanced CT would offer a significant clinical advantage. A potential barrier to clinical implementation is the time required for manual image segmentation. However, automated segmentation methods based on artificial intelligence may overcome this limitation and enable rapid calculation of GFR in clinical practice. Unlike iohexol plasma clearance testing, this methodology relies on the full iodinated contrast dose required for multiphasic CT, which may limit its applicability in patients with severe renal impairment.

There are several approaches to estimate renal function from imaging. The “Gates’ method” using radioisotopes provides reliable results but requires radiotracers and gamma camera equipment [7]. The “Patlak method” allows for GFR estimation using triphasic CT scans and dynamic contrast tracking but is mathematically complex [8, 9].

This study aimed to develop and evaluate a method to predict GFR from clinical renal CT by quantifying radiodensity and volume changes in the renal pelvis and bladder during unenhanced and excretory contrast phases and integrating them with age and body surface area (BSA).

## Materials and methods

### Patients

After ethical approval (Swedish Ethical Review Authority, Gothenburg, Sweden (Ref. 2023-00881-01)), candidates for living kidney donation between 2013 and 2022 or patients with muscle-invasive urinary bladder carcinoma who were candidates for neoadjuvant cisplatin prior to cystectomy between 2019 and 2024 were screened retrospectively. Both these groups routinely undergo renal CT and iohexol clearance testing as part of standard clinical workup. Informed consent was waived after IRB approval. Information was obtained from the electronic medical records and images extracted from the hospital’s Picture Archiving Communication System.

Body surface area (BSA) was calculated according to Du Bois [10]. mGFR (mL/min) was collected through iohexol clearance testing (mGFR_iohexol_) performed on the same day as the cystatin C and creatinine measurements. eGFR (mL/min) was derived from cystatin C (eGFR_cystatin C_) as well as creatinine using the Cockcroft–Gault formula (eGFR_Cockcroft-Gault_) and using the Revised Lund–Malmö formula (eGFR_LMR18_), which is based on both creatinine and cystatin C [11–13].

**Fig. 1:**
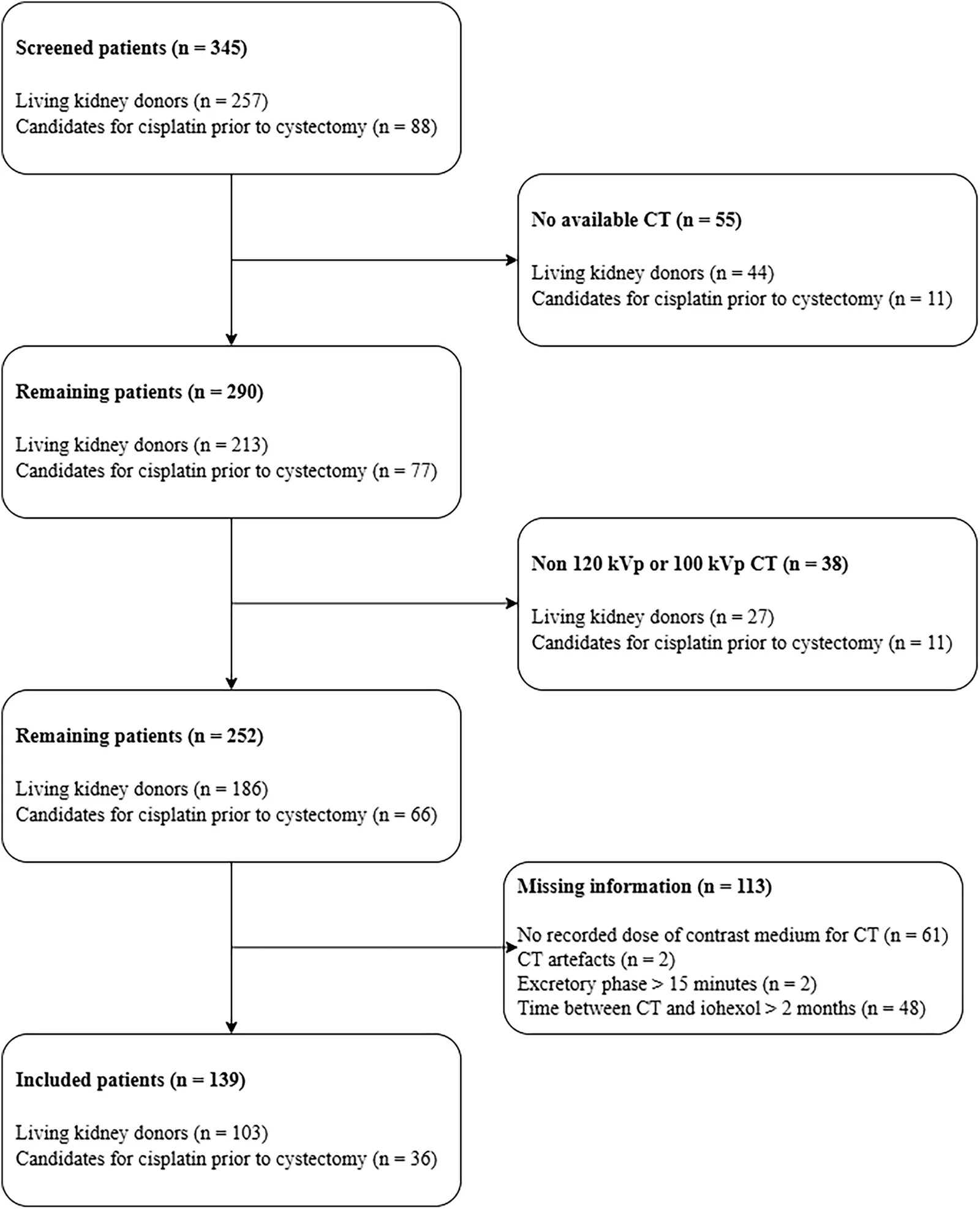
Flowchart of included and excluded study participants

Inclusion criteria were (1) a renal CT obtained at 120 or 100 kVp visualizing the renal pelvis and bladder before and after contrast administration, (2) an excretory phase CT image between 5–15 min after contrast administration, and (3) an iohexol clearance test performed within 2 months of the imaging and before surgery. Exclusion criteria were failure to meet inclusion criteria and improperly recorded amount of contrast (Fig. 1). Subjects examined using CT acquisition parameters outside the specified 100 and 120 kVp protocols were excluded.

### CT measurements

**Fig. 2:**
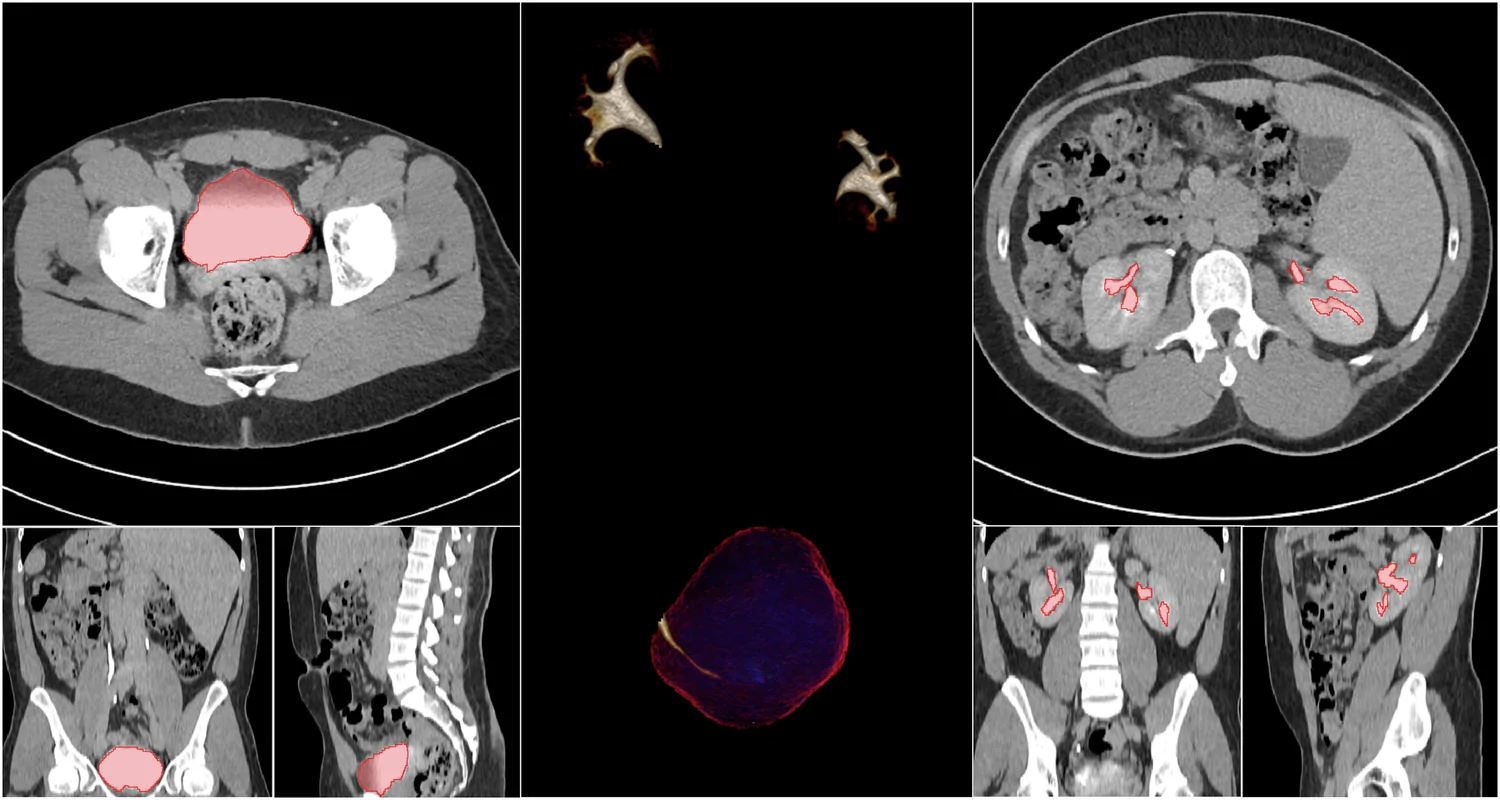
Segmentation of the renal pelvis and bladder in the excretory phase renal CT of a 46-year-old male patient

Renal pelvis and bladder were manually segmented from 5 mm thick axial images obtained in the unenhanced and excretory phases using ImageJ (National Institute of Health, version 1.53e) by a medical intern trained by a senior radiologist [14]. Volume and radiodensity (mean Hounsfield units (HU)) were recorded (Fig. 2). The excretory phase was defined as the delayed post-contrast CT acquisition, when excreted contrast was visible in the renal pelvis and urinary bladder. The CT_measurement_ was calculated using the formula:

$${{CT}}_{{measurement}}={ln}\left(\frac{\left(\left({{Volume}}_{{excretory}}* {{Radiodensity}}_{{excretory}}\right)-\left({{Volume}}_{{unenhanced}}* {{Radiodensity}}_{{unenhanced}}\right)\right)}{{{CM}}_{{mass}(g)}* {{time}}_{{injection\ excretory}}}\right)$$

The CT_measurement_ was designed as an imaging surrogate of the renal contrast excretion over the excretory period. The mean attenuation (HU) in contrast-enhanced urine is approximately proportional to iodine concentration. The difference in volume multiplied by respective HU yields an index proportional to the amount of excreted iodine.

We quantified net excreted contrast by the change in (HU × volume) from unenhanced to excretory phase (renal pelvis and bladder), normalized by the administered iodine dose (grams) and by the time from injection to excretory acquisition (minutes), yielding an index of excreted contrast per unit dose per unit time. To account for variation in excretory phase timing, CT_measurement_ was normalized by the time from contrast injection to excretory phase acquisition. The natural logarithm was applied to reduce right-skew and to linearize the association with mGFR_iohexol_ in linear regression.

At lower tube voltages, attenuation is more strongly influenced by the photoelectric effect. Because our cohort included acquisitions at both 100 and 120 kVp, we applied a normalization for HU values across the two protocols. HU values from 100 kVp examinations were scaled to an approximate 120 kVp equivalent, using a factor derived from published data, where the mean attenuation of iodinated fluid increased by 23% at 100 kVp compared with 120 kVp [15]. This represents an approximation rather than a physics-calibrated correction. To evaluate whether results were dependent on this assumption, we repeated the model development using only the 120 kVp subgroup.

### Automatic segmentation

The renal pelvis and urinary bladder were segmented in both the nonenhanced and excretory phases using *TotalSegmentator* (version 2.5.0), an open-weight U-Net-based neural network trained on CT scans from 1228 patients. All segmentations were visually inspected, corrected where necessary, and confirmed by a senior radiologist with 13 years of experience. The full automatic segmentation pipeline is described in the Supplementary Material.

Radiodensity values and volumes were then used to compute CT_measurement_, and a linear regression model was developed to predict mGFR_iohexol_, following the same procedure as for manual segmentation.

### Split renal function calculation (SRF)

Following segmentation of the left and right kidney using *TotalSegmentator* in both nonenhanced and excretory phases, the mean contrast accumulation in each kidney was calculated as the difference in mean radiodensity between the two phases, normalized by the average kidney volume across phases [16, 17].

$${Accumulation}= \left({{Radiodensity}}_{{excretory}}-{{Radiodensity}}_{{unenhanced}}\right) \\ \times \frac{({{Volume}}_{{excretory}}+{{Volume}}_{{unenhanced}})}{2}$$

SRF for the right kidney was then defined as the proportion of right kidney accumulation relative to total renal accumulation [16]. A lower threshold of −20 HU was applied to exclude fat surrounding the kidney and fat within the renal pelvis.

$${{Split}\,{renal}\,{function}}_{{\mbox{right}}}=\frac{{{Accumulation}}_{{\mbox{right}}}}{({{Accumulation}}_{{\mbox{right}}}+{{Accumulation}}_{{\mbox{left}}})}$$

### Statistical analysis

To determine GFR from renal CT scans (eGFR_CT_), multivariate linear regression analysis comparing mGFR_iohexol_ to age, BSA and the combined variable using CT_measurement_ was performed.

Because low mGFR_iohexol_ values were underrepresented in this retrospective cohort, reflecting the lower frequency of contrast-enhanced CT among patients with reduced renal function, we used weighted least squares to improve performance in the clinically important low-GFR range. Observations with mGFR_iohexol_ < 60 mL/min were assigned a higher weight (*n* = 7, weight = 10) representing a pragmatic compromise to increase their influence on model fitting without allowing this small subgroup to dominate the model. To evaluate the robustness of this choice, sensitivity analyses with alternative weights (weight = 5 and 15) were calculated for comparisons.

Agreement between mGFR_iohexol_ and eGFR_CT_ was assessed using Bland–Altman analysis by plotting the difference (mGFR_iohexol_ − eGFR_CT_) against the mean of the two methods. To evaluate proportional bias, we assessed the association between the differences and the mean values using linear regression and visually inspected the plot for non-constant variance. Agreement between manual and automatic segmentation was evaluated using a Bland–Altman analysis of the residuals and a paired Welch’s *t*-test to assess differences between residuals.

The mean absolute error (MAE) of eGFR_CT,_ eGFR_cystatin C_, eGFR_Cockcroft-Gault_, and eGFR_LMR18_ to the mGFR_iohexol_ was calculated separately. Statistical analyses were conducted using R Core Team (2013. R Foundation for Statistical Computing, version 4.3.3). A *p*-value of < 0.05 was considered statistically significant. The standard deviation (SD) is provided, if not otherwise stated.

Model performance was evaluated using repeated random split-sample validation. In each iteration, the dataset was split into a training group (80%) and a test group (20%) using stratified random sampling to preserve the proportion of donor and bladder-cancer patients in both sets. Performance metrics were computed on the test set and averaged across all iterations.

## Results

### Study population characteristics

139 patients out of 345 screened met the study criteria and none of the exclusion criteria (Fig. 1). Mean age was 52 years (± 13), and 60% were women (*n* = 83). Mean mGFR_iohexol_ was 99 mL/min (± 22), mean eGFR_cystatin C_ 100 mL/min (± 26), mean eGFR_Cockcroft-Gault_ 106 mL/min (± 30) and mean eGFR_LMR18_ 91 mL/min (± 17) (Table 1). The mean time between CT imaging and iohexol clearance test was 0.89 (± 0.57) months and between iohexol clearance test and cystatin C and creatinine was 0.1 (± 0.4) months. On average, 35.6 grams (IQR 32–40) iodine was administered at CT.

**Table 1:** Study population characteristics for included living kidney donors and patients considered for neoadjuvant cisplatin prior to cystectomy

Patient factor: mean (± SD)	Living kidney donors (*n* = 103)	Planned cystectomy (*n* = 36)
Age (years)	46.1 (± 10.7)	67.5 (± 7.1)
Sex		
Male	36 (35%)	20 (56%)
Female	67 (65%)	16 (44%)
Body surface area (m^2^)	1.85 (± 0.18)	1.92 (± 0.21)
mGFR_iohexol_ (mL/min)	104 (± 17)	83 (± 28)
eGFR_cystatin C_ (mL/min)	109 (± 20)	76 (± 24)
eGFR_creatinine_ (mL/min)		
Cockcroft–Gault	111 (± 26)	92 (± 33)
LMR18	94 (± 15)	81 (± 20)

*GFR* glomerular filtration rate, *LMR18* revised Lund–Malmö formula

**Fig. 3:**
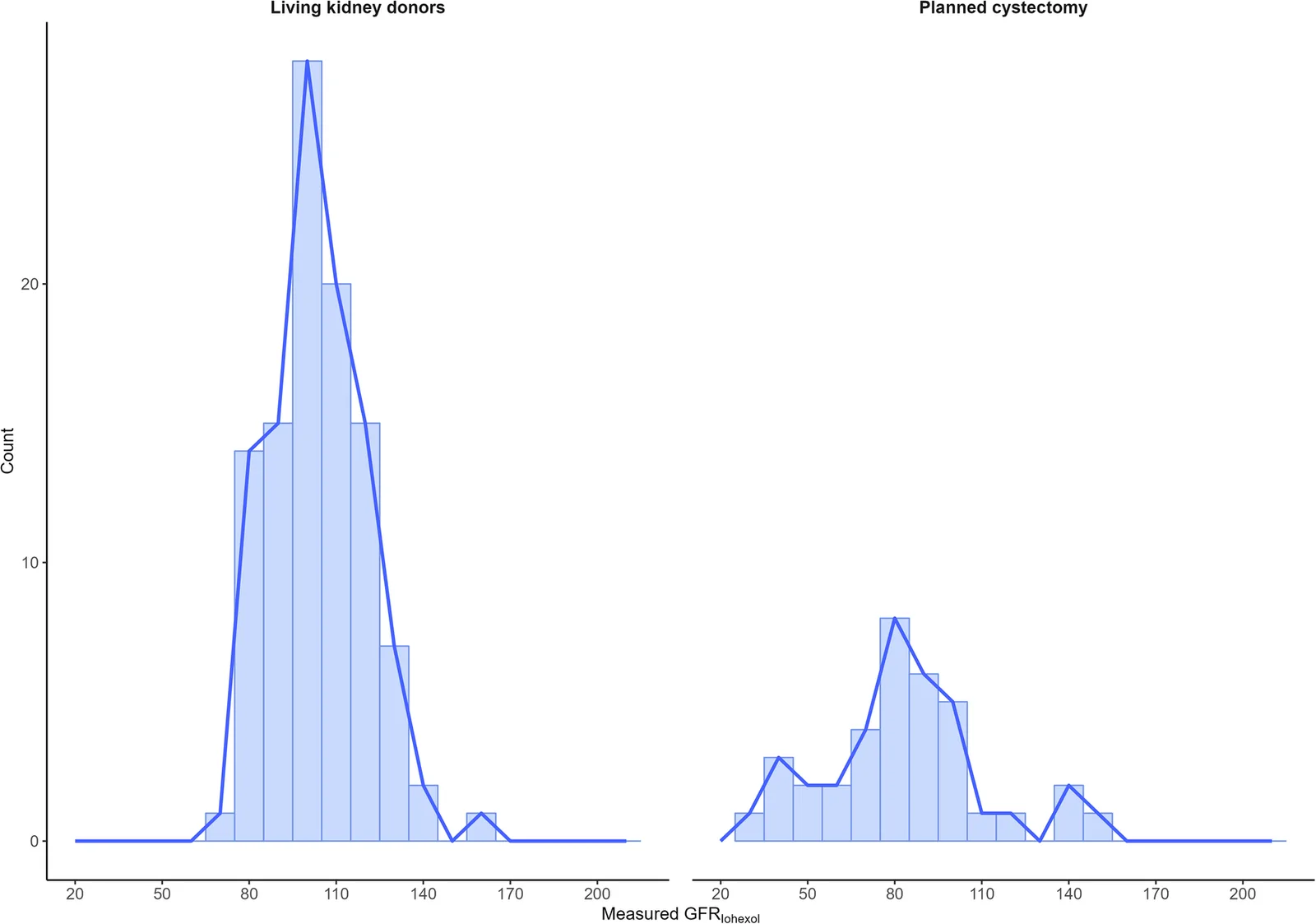
Distribution of measured glomerular filtration rate by iohexol plasma clearance in living kidney donors (*n* = 103) and patients planned for cystectomy (*n* = 36). Living kidney donors were predominantly represented at higher values, whereas patients planned for cystectomy had lower and more dispersed values, with partial overlap between the groups

Living kidney donors had higher mGFR_iohexol_ than patients planned for cystectomy, although there was overlap between the groups (Fig. 3).

### CT-derived eGFR

Multivariate linear regression analysis with mGFR_iohexol_ as a dependent variable and CT_measurement_, BSA and age as independent variables gave an adjusted R-squared of 0.77 (*p* < 0.001) with a Pearson correlation coefficient of 0.77.

$${{eGFR}}_{{ct}}=\left({{CT}}_{{measurement}}* 31.686\right)+\left({Age}* -1.103\right)\\ +\left({BSA}* 56.453\right)-124.537$$

The CT-derived estimate had a P10 of 40%, P20 of 80%, and P30 of 93%. Corresponding values were 45%, 73%, and 87% for eGFR_Cockcroft-Gault_, 40%, 77%, and 92% for eGFR_cystatin C_, and 45%, 81%, and 94% for eGFR_LMR18_.

**Fig. 4:**
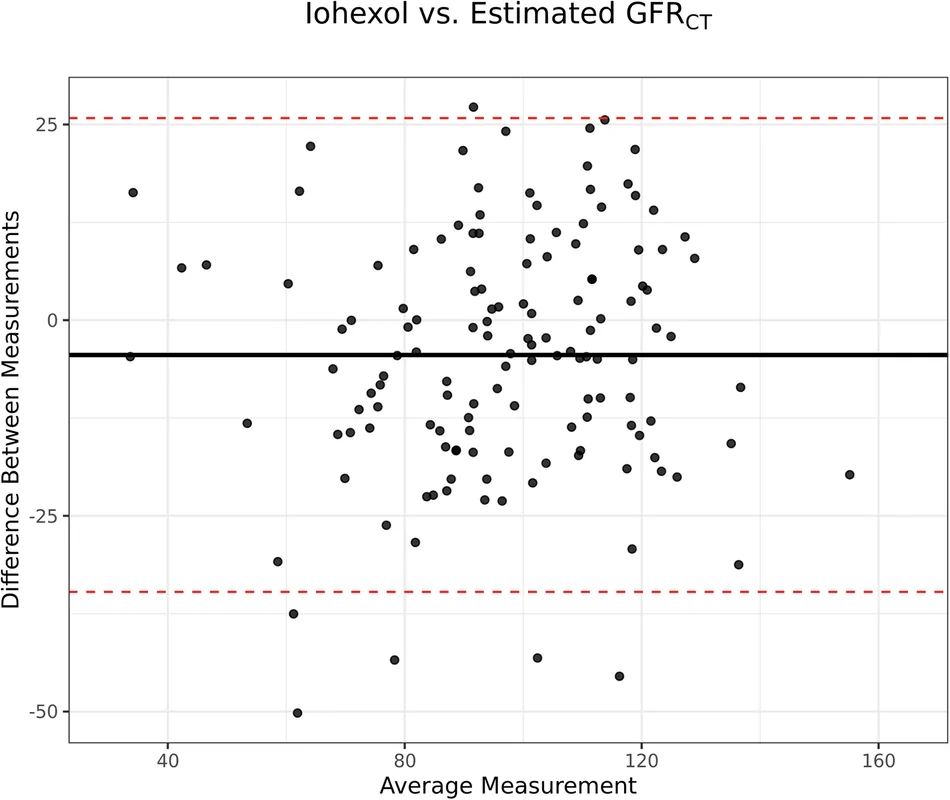
Bland–Altman plot comparing CT-estimated glomerular filtration rate eGFR_CT_ with mGFR from iohexol plasma clearance mGFR_iohexol_ in 139 patients. The mean difference (bias) was −4.4 mL/min, with 95% limits of agreement from −34.7 to 25.8

The underestimation of mGFR_iohexol_ by eGFR_CT_ was −4.4 mL/min (95% limits of agreement −34.7 to 25.8), without any evidence of proportional bias (Fig. 4).

**Fig. 5:**
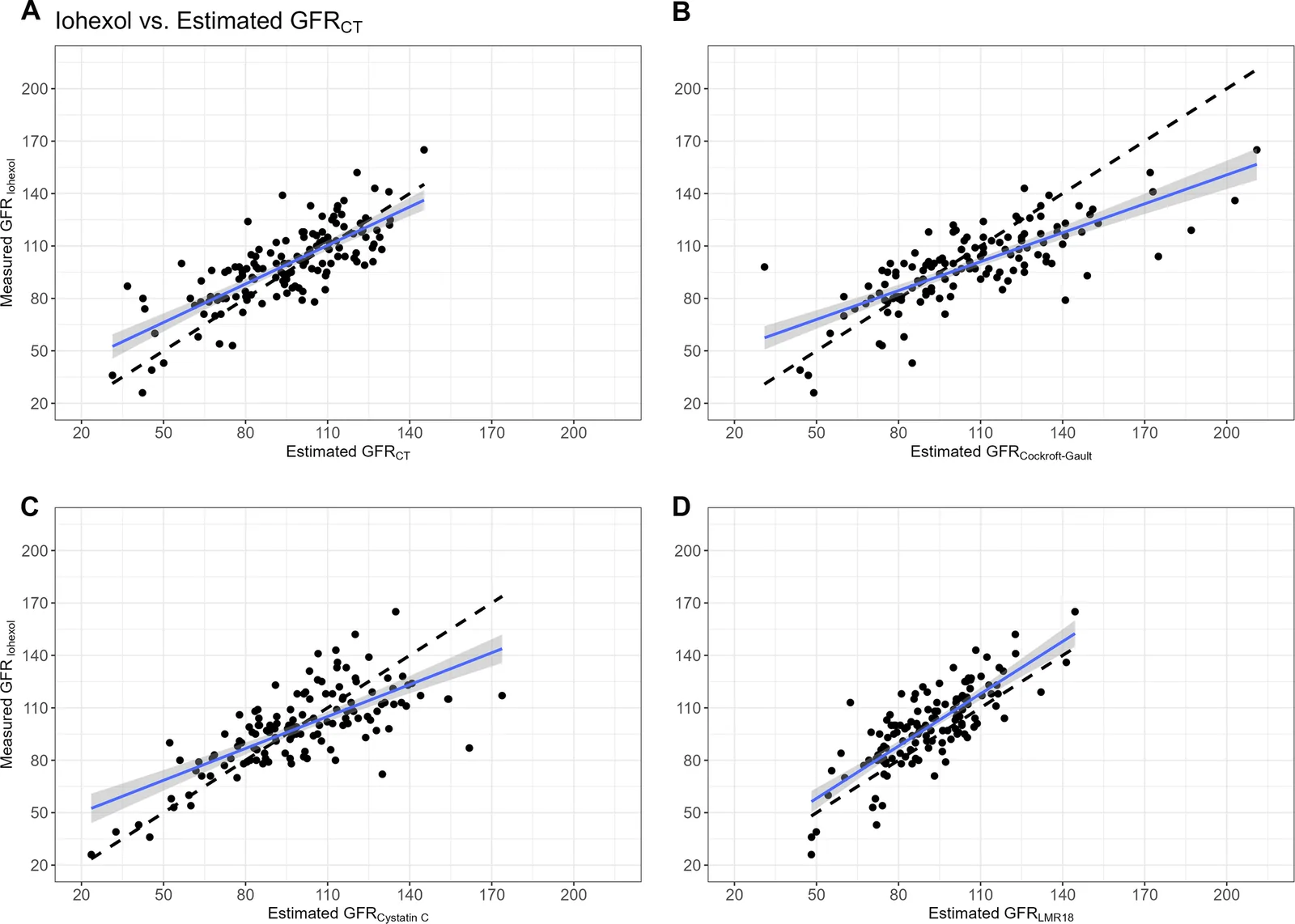
The relationship between different estimations of GFR (*x*-axis) and mGFR by iohexol plasma clearance (*y*-axis) in 139 patients. **A** The CT-derived estimate (eGFR_CT_), **(B)** Cockcroft–Gault eGFR, **(C)** cystatin C–based eGFR, and **(D)** Lund–Malmö formula–based eGFR. Black dots are individual participants. The solid blue line represents the fitted linear regression with 95% confidence interval. The dashed line denotes the line of identity (*y* = *x*)

eGFR_CT_ had the strongest relationship to mGFR_iohexol_ with a MAE of 12.8 mL/min. With eGFR_Cockcroft-Gault_, the MAE was 15.6 mL/min, for eGFR_cystatin C_, 14.2 mL/min, and for eGFR_LMR18_, 13.0 mL/min (Fig. 5).

The mean eGFR_CT_ for candidates for living kidney donation was 102.4 mL/min (± 17, *n* = 103) and for patients with bladder cancer eligible for neoadjuvant cisplatin prior to cystectomy, 71.4 mL/min (± 21, *n* = 36).

Varying the low-GFR weighting produced a similar model behavior. Using weight = 5 for mGFR_iohexol_ < 60 mL/min yielded an RMSE of 14.9 mL/min and MAE of 12.0 mL/min, while weight = 15 yielded an RMSE of 16.7 mL/min and MAE of 13.3 mL/min. Regression coefficients remained stable, with minimal variation in magnitude and consistent direction (Table 2).

**Table 2:** Different equations depending on weight used for values with GFR < 60 when constructing the ${{eGFR}}_{{ct}}$ calculation

Equation	Weight
${{eGFR}}_{{ct}}=\left({{CT}}_{{measurement}}* 31.0\right)+\left({{{\rm{Age}}}}* -1.0\right)+\left({{{\rm{BSA}}}}* 55\right)-115$	5
${{eGFR}}_{{ct}}=\left({{CT}}_{{measurement}}* 31.7\right)+\left({{{\rm{Age}}}}* -1.1\right)+\left({{{\rm{BSA}}}}* 56\right)-125$	10
${{eGFR}}_{{ct}}=\left({{CT}}_{{measurement}}* 31.1\right)+\left({{{\rm{Age}}}}* -1.0\right)+\left({{{\rm{BSA}}}}* 58\right)-129$	15

### Validation cohort and sensitivity analyses

The final full-cohort model showed better performance in living kidney donors compared to patients planned for cystectomy with RMSE 14.6 mL/min versus 19.6 mL/min, MAE 11.9 mL/min versus 15.4 mL/min, and P20 of 84% versus 67%.

There was little impact of the interval between CT and iohexol clearance testing, where patients with less than 30 days (*n* = 79) between the two examinations, instead of 60 days, showed an RMSE of 15.3 mL/min, MAE of 12.6 mL/min, and adjusted R² of 0.72.

To internally validate the model, we performed five iterations of stratified random split-sample validation. In each iteration, 80% of participants were randomly assigned to the training set and 20% to the test set. Across five runs, the mean RMSE was 17.1 mL/min and the mean MAE was 12.9 mL/min. The average Pearson correlation coefficient across the test sets was 0.73.

When model development and evaluation were repeated and restricted to examinations acquired at 120 kVp (*n* = 100), the model demonstrated similar performance compared with the full cohort, with an RMSE of 15.9 mL/min and MAE of 12.6 mL/min. Model fit remained high (R² = 0.798, adjusted R² = 0.792), and all predictors remained statistically significant.

### Automatic segmentation model

**Fig. 6:**
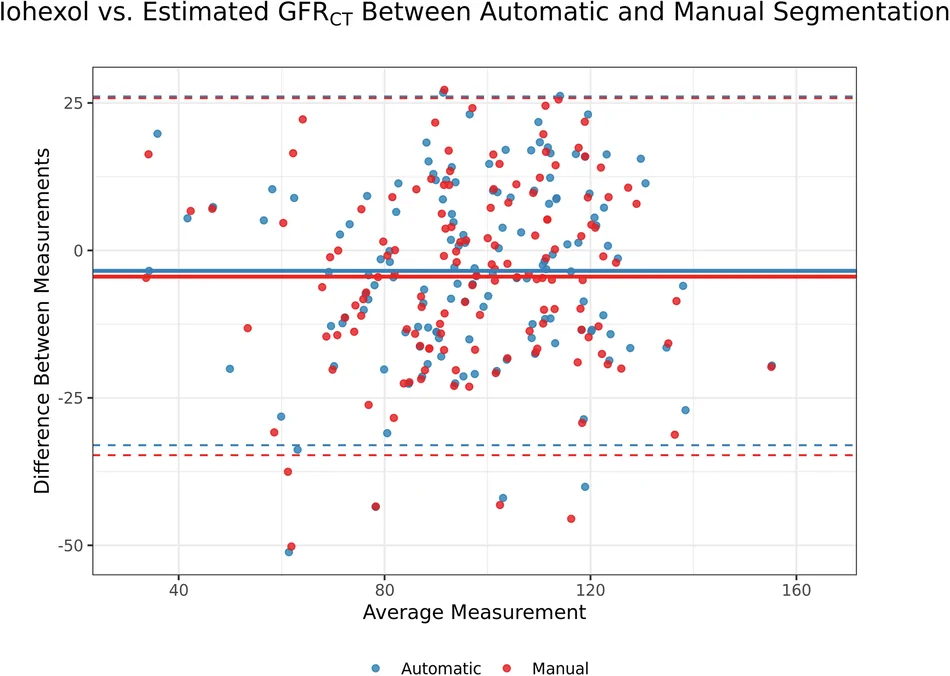
Bland–Altman plot comparing CT-estimated glomerular filtration rate eGFR_CT_ derived from automatic (blue) and manual (red) segmentation methods with mGFR from iohexol plasma clearance mGFR_iohexol_. The mean bias for the automatic method was −3.5 mL/min with 95% limits of agreement from −33.0 to 26.1. The mean bias for the manual method was −4.4 mL/min, with 95% limits of agreement from −34.7 to 25.8

Automatic segmentations required manual correction in 3 patients following visual review. Multivariate regression analysis using CT_measurement_ values from the automatic segmentations gave an adjusted R-squared value of 0.80 (*p* < 0.001), with an RMSE and MAE of 15.4 mL/min and 12.4 mL/min, respectively. The Bland–Altman analysis demonstrated similar limits of agreement and spread for the automatic and manual methods (Fig. 6).

### Split renal function analysis

The variability in SRF was substantially greater in the patients planned for cystectomy (0.577 ± 0.272) compared to the living kidney donors (0.487 ± 0.048), *p* < 0.0001, Supplementary Fig. 1.

## Discussion

Renal function could be estimated from routine, clinical renal CT with high precision by estimation of volume and radiodensity of the bladder and renal pelvis before and after contrast administration with adjustment for age and body surface area. Additionally, split renal function could be derived simultaneously without the need for additional imaging [17, 18], although not verified by renal scintigraphy in our study.

This method may be useful when both a precise estimate of kidney function and imaging of the kidneys are required, e.g., at living donor kidney assessments. More importantly, another potential use is the evaluation of deceased kidney donors. Currently, it is often impractical to perform an mGFR test, like an iohexol clearance test, due to time constraints. In clinical practice, risk-scoring systems and the donor’s most recent pre-donation serum creatinine are used to gauge organ quality [19]. However, elevated creatinine is frequently seen in deceased donors [20]. This can be attributed to reduced renal function, but also to factors such as muscle breakdown, liver disease, sepsis, and the use of medications that interfere with tubular secretion of creatinine [21].

Creatinine-based equations of eGFR have been shown to be subpar in patients with advanced liver disease, elevated bilirubin, or refractory ascites [22]. In the pretransplant evaluation for liver transplantation, the Model for End-stage Liver Disease (MELD) score is employed, incorporating renal function [23]. The CT-based GFR estimation could then be applied, both pre- and postoperatively, using their perioperative contrast-enhanced CT examinations. Likewise, patients undergoing nephrotoxic treatments, such as platinum-containing chemotherapy, would benefit from precise CT-based GFR measurements. The CT studies used to evaluate treatment effect can then be used to calculate the optimal treatment dose of renally excreted drugs such as carboplatin [24]. A similar rationale may apply to acute care settings in which rapidly changing renal function complicates drug dosing [25].

In 2023, Stehlé et al reported a method to calculate CT-GFR using iomeprol excretion rate change between arterial and excretory phases, normalized by aortic contrast HU [26]. While similar, our method uses the actual administered contrast dose rather than an indirect surrogate, reducing variability from vascular dynamics. Also, we did not segment according to thresholds, possibly better reflecting the excreted contrast. Additionally, by incorporating age and BSA, our model accounts for differences that influence renal clearance and contrast distribution. Finally, we achieved a lower mean absolute error (12.8 mL/min) compared to their ± 16 mL/min interquartile bias range.

GFR declines with age, independently of comorbidities [27]. Age not only correlates with declining GFR but also leads to a decrease in muscle mass accompanied by an increase in fat mass [28]. BSA was incorporated not only as a proxy for body habitus, which influences both GFR and contrast distribution, but also to account for technical artifacts such as beam hardening and photon starvation, which disproportionately affect image quality in individuals with higher body mass [29, 30].

As manual segmentation is time-consuming and exhibits limited reproducibility in routine clinical practice, automatic segmentation was investigated as an additional approach for predicting GFR. Similar predictive performance to the manual approach was achieved. Although three cases required manual correction, this likely reflects the use of a general-purpose open-weight model. Segmentation might be improved by task-specific neural network segmentation. The automatic kidney segmentation enables an approximate calculation of SRF. Patients planned for cystectomy had substantially greater variation in SRF compared to living kidney donors, probably due to asymmetric functional impairment associated with urinary bladder disease.

This study has several limitations. Renal function may have changed between CT and iohexol clearance testing. However, when restricting the censoring interval from 2 months to 1 month, no significant difference in model prediction was observed. The cohort also consisted of two highly selected clinical groups, with an underrepresentation of patients with moderate chronic kidney disease, resulting in a bimodal distribution of renal function. That may inflate correlation-based metrics such as R². We therefore emphasize absolute error metrics, and future studies should validate the method in larger cohorts with intermediate renal function ranges. In addition, no direct comparison with established imaging-based renal function methods, such as renal scintigraphy, was performed.

Our method assumes relatively normal urinary drainage and anatomy. Performance may therefore be affected in patients with urinary obstruction, marked anatomical anomalies, or neurogenic bladder. The correction applied for differences between 100 and 120 kVp is also approximate. Yet, even though kVp attenuation differences are influenced by patient size and beam-hardening effects and cannot be perfectly corrected with a single factor, similar results in the 120 kVp subgroup suggest that the main findings were not driven by this normalization. Phantom-based calibration would allow more precise comparison of HU values between 100 kVp and 120 kVp acquisitions. However, the predictive performance of our model across both settings underscores its potential applicability in clinical environments with varying imaging protocols. Finally, our method depends on the amount of contrast dose required for multiphasic renal CT, potentially limiting its use when reduced amounts are administered.

In conclusion, renal function can be estimated from standard renal CT with high precision. This model may be of special relevance in the evaluation of living and deceased kidney donors and for cancer patients who are candidates for nephrotoxic systemic therapies. In a larger perspective, eGFR_CT_ could be used in any clinical setting where a precise estimate of kidney function and multiphase contrast-enhanced abdominal CT is required.

## Supplementary information

ELECTRONIC SUPPLEMENTARY MATERIAL

## Data Availability

The data that support the findings of this study are available from the corresponding author upon reasonable request.

## Abbreviations

BSA: Body surface area
CT: Computed tomography
eGFR: Estimated glomerular filtration rate
GFR: Glomerular filtration rate
HU: Hounsfield units
MAE: Mean absolute error
mGFR: Measured glomerular filtration rate
RMSE: Root mean square error
SRF: Split renal function

## References

[1] Inker LA, Titan S (2021) Measurement and estimation of GFR for use in clinical practice: core curriculum 2021. Am J Kidney Dis 78:736–749 10.1053/j.ajkd.2021.04.016 34518032

[2] Speeckaert MM, Seegmiller J, Glorieux G et al (2021) Measured glomerular filtration rate: the query for a workable golden standard technique. J Pers Med 11:949 10.3390/jpm11100949 PMC8541429 34683089

[3] Delanaye P, Ebert N, Melsom T et al (2016) Iohexol plasma clearance for measuring glomerular filtration rate in clinical practice and research: a review. Part 1: How to measure glomerular filtration rate with iohexol? Clin Kidney J 9:682–699 10.1093/ckj/sfw070 PMC5036902 27679715

[4] Chen DC, Potok OA, Rifkin D, Estrella MM (2022) Advantages, limitations, and clinical considerations in using cystatin C to estimate GFR. Kidney360 3:1807–1814 10.34067/KID.0003202022 PMC9717651 36514729

[5] Porrini E, Ruggenenti P, Luis-Lima S et al (2019) Estimated GFR: time for a critical appraisal. Nat Rev Nephrol 15:177–190 10.1038/s41581-018-0080-9 30518813

[6] Alaini A, Malhotra D, Rondon-Berrios H et al (2017) Establishing the presence or absence of chronic kidney disease: uses and limitations of formulas estimating the glomerular filtration rate. World J Methodol 7:73–92 10.5662/wjm.v7.i3.73 PMC5618145 29026688

[7] You S, Ma X, Zhang C et al (2018) Determination of single-kidney glomerular filtration rate (GFR) with CT urography versus renal dynamic imaging Gates method. Eur Radiol 28:1077–1084 10.1007/s00330-017-5061-z 28971295

[8] Tsushima Y (1999) Functional CT of the kidney. Eur J Radiol 30:191–197 10.1016/s0720-048x(99)00011-x 10452717

[9] Yuan X, Zhang J, Tang K et al (2017) Determination of glomerular filtration rate with CT measurement of renal clearance of iodinated contrast material versus ^99m^Tc-DTPA dynamic imaging “Gates” method: a validation study in asymmetrical renal disease. Radiology 282:552–560 10.1148/radiol.2016160425 27556274

[10] Du Bois D, Du Bois EF (1989) A formula to estimate the approximate surface area if height and weight be known. 1916. Nutrition 5:303–311 2520314

[11] Björk J, Nyman U, Delanaye P et al (2020) A novel method for creatinine adjustment makes the revised Lund-Malmö GFR estimating equation applicable in children. Scand J Clin Lab Invest 80:456–463 10.1080/00365513.2020.1774641 32628043

[12] Cockcroft DW, Gault MH (1976) Prediction of creatinine clearance from serum creatinine. Nephron 16:31–41 10.1159/000180580 1244564

[13] Grubb A, Horio M, Hansson LO et al (2014) Generation of a new cystatin C-based estimating equation for glomerular filtration rate by use of 7 assays standardized to the international calibrator. Clin Chem 60:974–986 10.1373/clinchem.2013.220707 24829272

[14] Schneider CA, Rasband WS, Eliceiri KW (2012) NIH Image to ImageJ: 25 years of image analysis. Nat Methods 9:671–675 10.1038/nmeth.2089 PMC5554542 22930834

[15] Chang KJ, Caovan DB, Grand DJ, Huda W, Mayo-Smith WW (2013) Reducing radiation dose at CT colonography: decreasing tube voltage to 100 kVp. Radiology 266:791–800 10.1148/radiol.12120134 23264348

[16] Summerlin AL, Lockhart ME, Strang AM, Kolettis PN, Fineberg NS, Smith JK (2008) Determination of split renal function by 3D reconstruction of CT angiograms: a comparison with gamma camera renography. AJR Am J Roentgenol 191:1552–1558 10.2214/AJR.07.4023 PMC2701148 18941100

[17] Frennby B, Almén T, Lilja B et al (1995) Determination of the relative glomerular filtration rate of each kidney in man. Comparison between iohexol CT and 99mTc-DTPA scintigraphy. Acta Radiol 36:410–417 7619622

[18] Lal H, Singh A, Prasad R et al (2021) Determination of split renal function in voluntary renal donors by multidetector computed tomography and nuclear renography: How well do they correlate? SA J Radiol 25:2009 10.4102/sajr.v25i1.2009 PMC8008088 33824742

[19] Rao PS, Schaubel DE, Guidinger MK et al (2009) A comprehensive risk quantification score for deceased donor kidneys: the kidney donor risk index. Transplantation 88:231–236 10.1097/TP.0b013e3181ac620b 19623019

[20] Hall IE, Schröppel B, Doshi MD et al (2015) Associations of deceased donor kidney injury with kidney discard and function after transplantation. Am J Transplant 15:1623–1631 10.1111/ajt.13144 PMC4563988 25762442

[21] Ostermann M, Joannidis M (2016) Acute kidney injury 2016: diagnosis and diagnostic workup. Crit Care 20:299 10.1186/s13054-016-1478-z PMC5037640 27670788

[22] Gonwa TA, Jennings L, Mai ML, Stark PC, Levey AS, Klintmalm GB (2004) Estimation of glomerular filtration rates before and after orthotopic liver transplantation: evaluation of current equations. Liver Transpl 10:301–309 10.1002/lt.20017 14762871

[23] Aranzana EM, Coppini AZ, Ribeiro MA et al (2015) Model for end-stage liver disease, model for liver transplantation survival and donor risk index as predictive models of survival after liver transplantation in 1,006 patients. Clinics (Sao Paulo) 70:413–418 10.6061/clinics/2015(06)05 PMC4462569 26106959

[24] Calvert AH, Newell DR, Gumbrell LA et al (1989) Carboplatin dosage: prospective evaluation of a simple formula based on renal function. J Clin Oncol 7:1748–1756 10.1200/JCO.1989.7.11.1748 2681557

[25] Chen IH, Nicolau DP (2020) Augmented renal clearance and how to augment antibiotic dosing. Antibiotics 9:393 10.3390/antibiotics9070393 PMC7399877 32659898

[26] Stehlé T, Wei F, Brabant S et al (2023) Glomerular filtration rate measured based on iomeprol clearance assessed at CT urography in living kidney donor candidates. Radiology 309:e230567 10.1148/radiol.230567 38085083

[27] Guppy M, Thomas ET, Glasziou P et al (2024) Rate of decline in kidney function with age: a systematic review. BMJ Open 14:e089783 10.1136/bmjopen-2024-089783 PMC11603750 39609029

[28] Evans WJ, Campbell WW (1993) Sarcopenia and age-related changes in body composition and functional capacity. J Nutr 123:465–468 10.1093/jn/123.suppl_2.465 8429405

[29] Ginde AA, Foianini A, Renner DM, Valley M, Camargo CA Jr (2008) The challenge of CT and MRI imaging of obese individuals who present to the emergency department: a national survey. Obesity (Silver Spring) 16:2549–2551 10.1038/oby.2008.410 18787528

[30] Schindera ST, Nelson RC, Toth TL et al (2008) Effect of patient size on radiation dose for abdominal MDCT with automatic tube current modulation: phantom study. AJR Am J Roentgenol 190:W100–W105 10.2214/AJR.07.2891 18212190

