# Supplementary material for: Estimation of glomerular filtration rate by standard renal CT imaging
Source: Insights Imaging. 2026 Oct 14;17:257. doi: 10.1186/s13244-026-02431-0 (PMC13669286; doi:10.1186/s13244-026-02431-0)
Supplement: Supplementary file 1 [file 13244_2026_2431_MOESM1_ESM.pdf]

Estimation of Glomerular Filtration Rate by Standard Renal CT Imaging

ELECTRONIC SUPPLEMENTARY MATERIAL

Automatic Segmentation of Renal Pelvis and Urinary Bladder

All CT images were converted from DICOM to NIfTI format and subsequently processed using TotalSegmentator v2 to segment the left kidney, right kidney, and urinary bladder. All segmentations were visually inspected in axial, sagittal, and coronal planes.

To delineate the renal pelvis, a convex hull operation was applied to each kidney to include the renal pelvic region within the kidney mask. For the excretory phase, a high-HU threshold (>300 HU) was used to isolate contrast-enhanced urine, and the largest connected component was retained as the renal pelvis. All renal pelvis segmentations were visually inspected in axial, sagittal, and coronal planes, and no manual corrections were necessary. For the non-enhanced phase, the renal pelvis volume was assumed to be equivalent to that of the excretory phase, with an assumed mean attenuation of 0 HU.

Fig S1: Histogram of split renal function between living donor patients (blue, n=103) and patients planned for cystectomy (red, n=36). Living kidney donors had a smaller variance compared to patients planned for cystectomy.

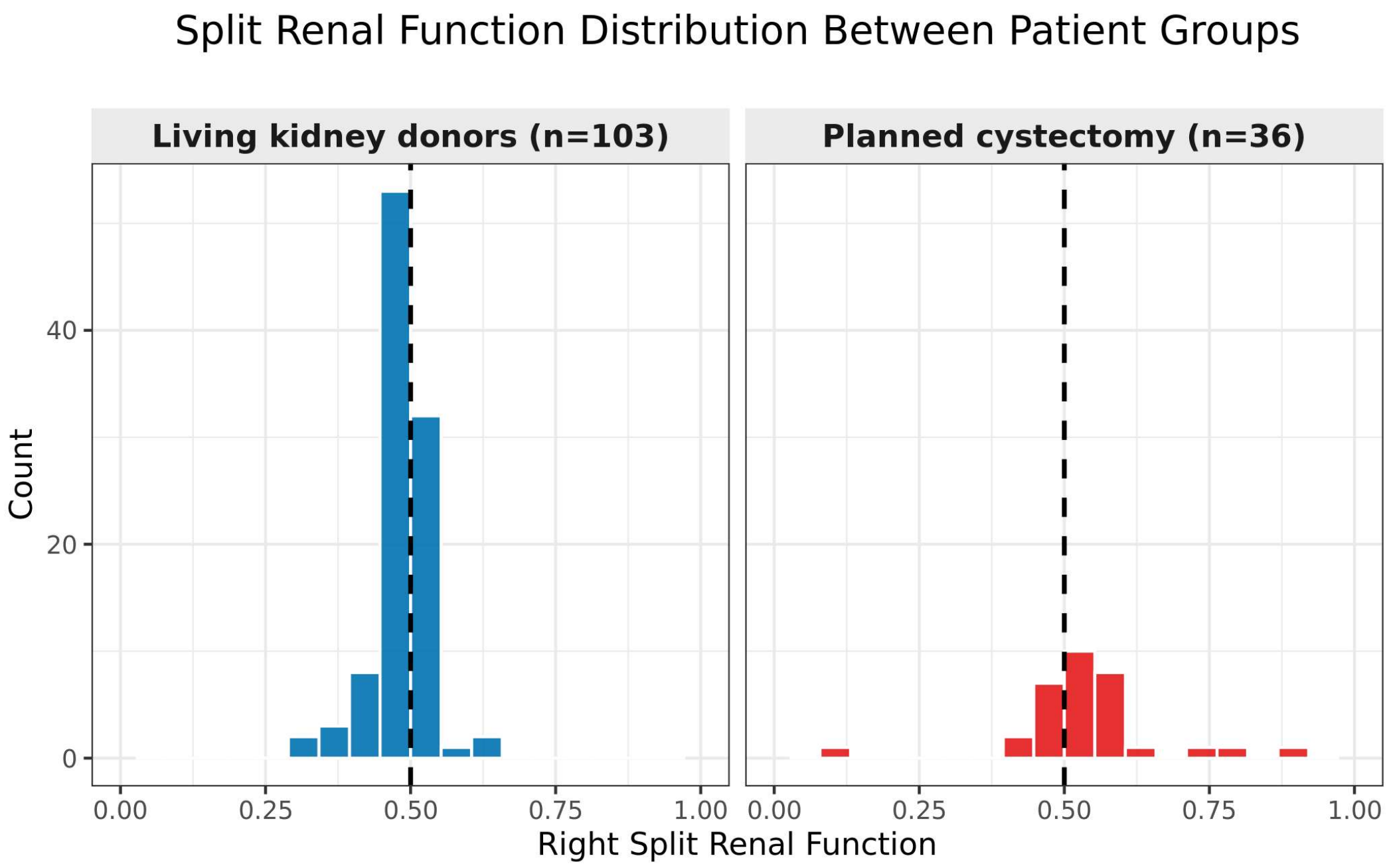

Insights Imaging (2026) Huitfeldt Sola K, Genberg H, Webster-Ford J, Ullén A, Brismar T.
